# The Neural Crest as the First Production Site of the Erythroid Growth Factor Erythropoietin

**DOI:** 10.3389/fcell.2019.00105

**Published:** 2019-06-12

**Authors:** Ikuo Hirano, Norio Suzuki

**Affiliations:** ^1^Department of Molecular Hematology, Graduate School of Medicine, Tohoku University, Sendai, Japan; ^2^Division of Oxygen Biology, United Centers for Advanced Research and Translational Medicine, Graduate School of Medicine, Tohoku University, Sendai, Japan

**Keywords:** erythropoietin, hypoxia, erythropoiesis, reporter mice, REP cell

## Abstract

While the neural crest is considered the fourth germ layer that originates a variety of tissues during mammalian development, we recently discovered that some neural crest cells and neuroepithelial cells play a unique role in secreting a vital hematopoietic hormone, erythropoietin (EPO), in mouse embryos. EPO production by the neural crest is transient in mid-stage embryos but essential for the first erythropoiesis in the yolk sac and for sufficient oxygen supply in the whole embryo growing *in utero*. The site of EPO production shifts from the neural crest to the liver in late embryonic stages, followed by interstitial fibroblasts of the kidneys in adults. Interestingly, the transition of EPO production sites synchronizes with the transition of erythropoietic sites during mouse development from the yolk sac and the fetal liver to the bone marrow. EPO produced by the neural crest and the neuroepithelium is first stored around the production sites and delivered to the yolk sac as an endocrine hormone for erythropoiesis after heartbeat activation. The fact that EPO is produced by some human cell lines derived from neuroblastoma, which mainly originates from the neural crest, provides evidence that the neural crest secretes EPO for primitive erythropoiesis not only in mouse but also in human embryos. Here, we introduce and discuss recent progress in studies on the mechanism of EPO production by the neural crest and its roles in erythropoietic development and in the fate of EPO-producing neural crest cells.

## Introduction

Red blood cells (RBCs) are required to deliver oxygen, which is a vital molecule for aerobic respiration, into every organ from the lung via the circulation system in adult mammals. Therefore, the production of RBCs (erythropoiesis) is provoked under low oxygen conditions (hypoxia) by erythropoietin (EPO), which is the essential erythroid growth factor produced by the kidney in a hypoxia-inducible manner (Suzuki, 2015; Suzuki and Yamamoto, 2016). EPO induces proliferation, differentiation and survival of erythroid progenitor cells through binding to its specific receptor, the EPO receptor (EPOR), which is expressed on the surface of those cells (Nandakumar et al., 2016). EPO secretion from the kidney is fundamentally regulated at the transcriptional level of the *EPO* gene in renal EPO-producing (REP) cells, which are fibroblastic cells localized in the interstitium of the renal cortex (Suzuki and Yamamoto, 2016).

In early embryonic stages, low-level oxygen is directly diffused into embryos, and the hypoxic environment in developing embryos has been correlated with stem-cell pluripotency and morphogenesis (Ezashi et al., 2005). In fact, a gradient in oxygen concentrations in chicks is important for normal morphogenesis (Giussani et al., 2007; Lourens et al., 2007). Mid- to late-stage embryos, in which many cells are aggressively proliferating, require an efficient and constant oxygen supply governed by RBCs and the circulation system. To stimulate embryonic erythropoiesis, EPO is produced by the hepatocytes of fetuses, in which the kidneys are under construction. Therefore, the loss of EPO production in the mouse fetal liver results in embryonic death due to severe anemia (Wu et al., 1995; Yamazaki et al., 2013).

During mammalian development, erythropoiesis is active before hepatocytes initiate EPO production (Wu et al., 1995; Suzuki et al., 2013). We discovered that the first site of EPO production in mouse embryos is a portion of the neural crest cells and neuroepithelial cells, which have been referred to as neural EPO-producing (NEP) cells (Malik et al., 2013; Suzuki et al., 2013). As the neural crest is an embryo-specific tissue, EPO production by NEP cells is transient in mid-embryonic stages. EPO secreted by NEP cells is delivered to erythroid progenitor cells in the yolk sac and bloodstream to support embryonic erythropoiesis (Suzuki et al., 2013). However, the fate of NEP cells and the regulatory mechanisms of *EPO*-gene expression have not been fully elucidated. In this chapter, we introduce the newly identified role of neural crest cells in the production of the essential erythroid growth factor for the oxygenation of whole embryos. In addition, features of NEP cells are discussed.

## Erythropoiesis During Mouse Development

**FIGURE 1:**
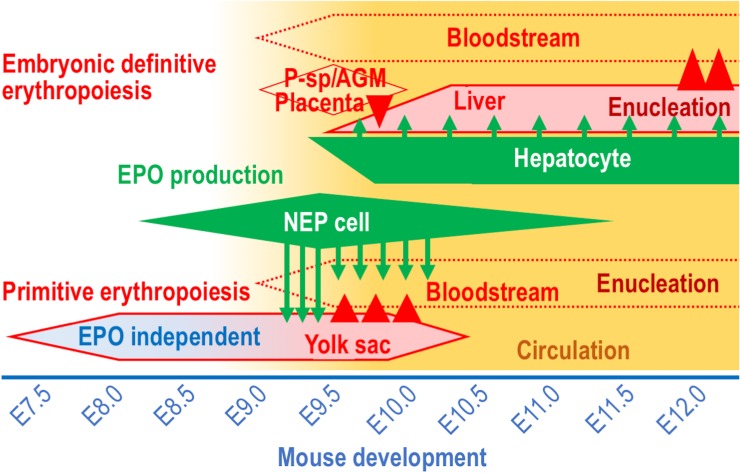
The EPO-producing cells and the erythropoietic sites in mouse development. The sites of EPO production (green) and erythropoiesis (red) during mouse development between E7.5 and E12.0. Circulation (yellow) is gradually activated around E9.0. Arrows and arrowheads indicate EPO secretion and cell migration, respectively. Note that the primitive erythroid cells are generated in the yolk sac in an EPO-independent manner until the initiation of active circulation.

Developing organs require much oxygen for energy production in mammalian embryos, and hemoglobins in RBCs are essential oxygen couriers that consist of globin proteins and heme. To effectively deprive maternal hemoglobins of oxygen in the placenta, the oxygen affinity of embryonic hemoglobins is higher than that of adult hemoglobins. During mouse development, the production of RBCs carrying embryonic hemoglobins is initiated in the blood islands of visceral yolk sacs around embryonic day (E) 7.5, and these blood islands are Milky Way-like cell clusters containing both hematopoietic and endothelial progenitors (Palis, 2014; Figure 1). RBCs grown in the blood islands are gradually released into the bloodstream after E9.0, when the circulation system begins to work. While the adult “definitive” RBCs emerge from hematopoietic sites in the bone marrow (and in the spleen for rodents) into the bloodstream after terminal maturation and nuclear removal (enucleation, Figure 1), the “primitive” RBCs from the yolk sac are discharged into the bloodstream before enucleation and terminally maturate during circulation (Palis, 2014). Although the growth of erythroid cells in the yolk sac is transient and terminates at approximately E10.0, the maturation of the primitive erythroid cells continues in the bloodstream until later embryonic stages (Lux et al., 2008).

In mammalian embryos, the major erythropoietic site transfers from the yolk sac to liver, into which multipotential hematopoietic progenitor cells migrate from the paraaortic splanchnopleura (P-Sp)/aorta-gonad-mesonephros (AGM) region, placenta and yolk sac until E9.5 (McGrath and Palis, 2008; Figure 1). The multipotential hematopoietic progenitors proliferate, and some of the cells differentiate into erythroid-specific progenitors in the fetal liver. RBCs grown in the fetal liver undertake enucleation on site and enter the bloodstream after E12.0 (Kingsley et al., 2004; Figure 1). Thus, throughout mammalian life, there are 3 erythropoietic waves, the yolk sac primitive erythropoiesis, the fetal liver definitive erythropoiesis and the adult definitive erythropoiesis, which are distinguished from each other based on the size of the RBCs, the oxygen affinity of hemoglobins and the sites of enucleation (Palis, 2014).

## REP Cells: the Major Site of Erythropoietin Production in Adults

EPO is a 34-kDa glycoprotein that is highly conserved among mammals as an essential erythroid growth factor. EPO binding to EPOR on the surface of erythroid precursor cells conveys signals for proliferation, differentiation and anti-apoptosis, mainly by activating the JAK2-STAT5 pathway (Elliott and Sinclair, 2012; Kuhrt and Wojchowski, 2015). Target genes of the transcription factor STAT5, which is phosphorylated and activated by EPO signaling, include the genes for erythroferrone (a regulator of iron metabolism), transferrin receptor (an iron intake transporter), Bcl-xL (an anti-apoptotic factor), and *CIS* (a mediator of EPO signaling) (Kuhrt and Wojchowski, 2015; Suzuki and Yamamoto, 2016).

In adult mice, EPO production levels in the kidney are enhanced under hypoxic conditions. The liver and brain also produce EPO in a hypoxia-inducible manner, but at least 80% of serum EPO is thought to be derived from the kidney (Weidemann and Johnson, 2009). In fact, *EPO*-gene expression levels in the liver and brain are very low compared to those in the kidney (Obara et al., 2008). Additionally, kidney damage often causes renal anemia as a complication of chronic kidney disease (Nangaku and Eckardt, 2006). Among a variety of cell types in the kidney, a specific fraction of interstitial fibroblasts (referred to as REP cells) is responsible for EPO production. REP cells are mainly located in the renal cortex and corticomedulla boundary regions (Yamazaki et al., 2013). Interestingly, the expression of neural genes, such as *Ngfr* (encoding p75^NTR^), *Map2* (encoding microtubule associated protein 2) *Nefl* (encoding neurofilament light chain), *Nes* (encoding nestin) and *Cspg4* (encoding neuron-glial antigen 2), is detectable in REP cells (Obara et al., 2008; Asada et al., 2011; Imeri et al., 2019; ÓhAinmhire et al., 2019). REP-cell precursors are detectable in developing kidneys at E13.5, and these cells express the *Epo* gene in late embryonic stages (Asada et al., 2011; Pan et al., 2011; Suzuki et al., 2011). Although the extrarenal origin of REP cells has been proposed (Asada et al., 2011; Matsumoto et al., 2012), the developmental origin of REP cells is controversial.

In REP cells, the rate-limiting step of EPO production is the transcriptional regulation of the *Epo* gene by the hypoxia-inducible transcription factor 2α (HIF2α), which is degraded under normal air conditions and stabilized by hypoxic stress (Souma et al., 2016). HIF2α-inducible *Epo*-gene expression is critical to maintain systemic oxygen homeostasis through erythropoietic regulation. For REP-cell-specific EPO production, the renal enhancer sequence located 17.4- to 3.6-kb upstream of the mouse *Epo* gene transcriptional start site is essential, while hepatic EPO production requires the proximal downstream region (Suzuki et al., 2011; Suzuki, 2015; Hirano et al., 2017).

## Identification of Cells Expressing the *EPO* Gene in Developing Mouse Embryos

In addition to adult erythropoiesis requiring EPO, the EPO-EPOR pathway is essential for erythropoiesis in embryos. In fact, mice lacking either EPO or EPOR exhibit embryonic lethality due to severe anemia at approximately E13.5 when the liver produces both RBCs and EPO (Wu et al., 1995; Suzuki et al., 2013). For embryonic definitive erythropoiesis in the liver, EPO produced by hepatocytes in a paracrine manner stimulates the growth of erythroid cells inside the liver (Suzuki et al., 2011). The hepatocytes maintain EPO production in adults, but their contribution to adult erythropoiesis is very minor compared to the EPO production from REP cells (Suzuki et al., 2011).

At E9.0, before the hepatocytes express the *Epo* gene, the expression levels of the transferrin receptor on the cell surface of erythroid cells are reduced in EPO-knockout embryos compared to those in wild-type embryos (Suzuki et al., 2013). Then, at E10.5–E11.5, the primitive erythroid cells in mouse embryos are decreased by EPO deficiency (Wu et al., 1995; Suzuki et al., 2013). These observations indicate that EPO production in E9.0 embryos is required for normal primitive erythropoiesis in the yolk sac and bloodstream. However, because complete loss of primitive erythropoiesis in mouse embryos results in lethality around E9.5 (Palis, 2014), survival of EPO knockout embryos until E13.5 suggests that the “partial” defect of primitive erythropoiesis due to EPO deficiency is unrelated to mouse mortality.

To apparently identify EPO-producing sites, we adopted gene-modified mouse lines expressing green fluorescent protein (GFP) under the control of mouse *Epo* gene regulatory regions. Analyzing many lines of the reporter transgenic mice with a variety of gene regulatory regions, the *Epo*-gene flanking region ranging from 17.4 kb upstream to the proximal downstream of the *Epo*-gene coding region is needed to faithfully recapitulate the endogenous *Epo* gene expression profile in mice (Hirano et al., 2017). Indeed, the GFP transgenes are expressed in REP cells and hepatocytes of adult mice in a hypoxia/anemia-inducible manner (Obara et al., 2008; Suzuki et al., 2011; Hirano et al., 2017). Interestingly, the transgenic mice revealed that EPO is also produced by a small population of glial fibrillary acidic protein (GFAP)-positive astrocytes in the adult brain under hypoxic conditions.

**FIGURE 2:**
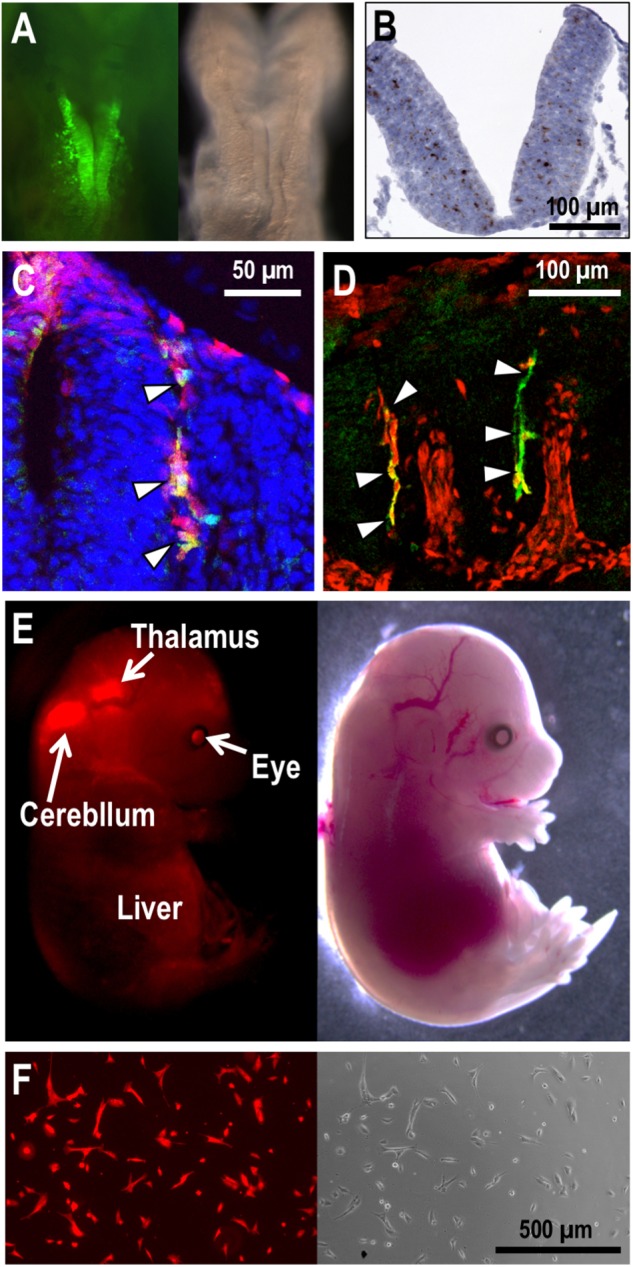
NEP cells and their derivatives in mouse embryos. **(A)** *EpoGFP* transgene expression (green) around the neural fold of an E8.5 embryo. Fluorescent (left) and bright-field (right) images are shown. **(B)** *Epo* mRNA expression (brown) in the neural tube of an E9.5 embryo. Hematoxylin was used to counterstain the section applied for *in situ* hybridization. **(C,D)** *Cre* transgenes expressed under the *Wnt1*- **(C)** and *Mpz*- **(D)** gene regulation label the neural crest cells with tdTomato reporter expression (red) in mouse embryos at E9.5 **(C)** and E10.5 **(D)**. *EpoGFP* transgene expression (green) is detected in tdTomato-positive neural crest cells (arrowheads) migrating from dorsal (upper) to ventral (lower) regions in the intersomatic spaces. **(E)** Tracing the cell fate of NEP cells in mouse embryos. *EpoCre* transgene-mediated tdTomato expression (red) traces the cell fate of NEP cells in an E15.5 embryo. Fluorescent (left) and bright-field (right) images are shown. **(F)** The Neplic cell line established from the tdTomato-positive brain cells of the embryo shown in **E**.

In embryos bearing the *EpoGFP* transgene, GFP expression is detected in the hepatocytes as expected, and the hepatocyte precursors (hepatoblasts) budding in the ventral trunk region also express GFP (Suzuki et al., 2013; Hirano et al., 2017). While hepatic GFP expression is detectable in the embryos after E9.5, a small number of rostral neural cells initiate GFP expression at E8.5 (Suzuki et al., 2013; Figure 2A). The GFP-positive NEP cells expand and distribute in and around the neural tube. NEP cells are considered the developmentally first source of EPO for primitive erythropoiesis. In fact, the first *Epo* mRNA expression is detectable in the embryonic proper but not in the yolk sac and placenta at E8.5 (Suzuki et al., 2013; Palis, 2014).

The *Epo* gene expression profile in NEP cells, which has been identified by multiple transgenic mouse lines bearing the various *EpoGFP* transgenes, was confirmed by gene-modified mouse embryos, in which the endogenous *Epo*-gene coding region was replaced with the *GFP* cDNA (Suzuki et al., 2013; Hirano et al., 2017). Additionally, an advanced *in situ* hybridization technique (RNAscope established by ACD Inc.) precisely and directly detected the endogenous *Epo* mRNA expression in NEP cells of mid-stage mouse embryos (Figure 2B). Although *Epo* mRNA expression in the vitelline vessel and urogenital ridge of E9.0–E10.0 mouse embryos was reported (Lee et al., 2001), the GFP reporter mouse system, the *in situ* hybridization technique and quantitative RT-PCR in our hands hardly demonstrated *Epo*-gene expression in these tissues (Suzuki et al., 2013). Therefore, we suggest that the improved detection technologies identify embryonic *Epo*-gene expression with high sensitivity, high quantitativity, high resolution (at the cellular level) and low noise.

## Roles of EPO Derived From NEP Cells

As mentioned above, the GFP-labeled NEP cells emerge on the neural fold at E8.5, and then, the cells are explosively increased and distributed into the neural tube, optic vesicle, otic vesicle and intersomatic spaces, all of which are derived from the neural crest (Suzuki et al., 2013). Transgenic mouse lines bearing neural crest cells and neural crest-derived cells permanently labeled with tdTomato fluorescent protein expression have demonstrated that the *EpoGFP* transgenes are expressed in a portion of neural crest cells (Lee et al., 1997; Jiang et al., 2000; Suzuki et al., 2013; Figure 2C,D). NEP cells are distributed not only into the neural crest and its derivative tissues but also into the neuroepithelium of embryos defined by the expression of the neuronal transcription factor SOX2 (Suzuki et al., 2013). Endogenous *Epo*-gene expression in NEP cells was confirmed by means of RT-PCR using isolated GFP-positive cells and *in situ* hybridization (Figure 2B). Thus, NEP cells in the neural crest, neural crest-derived tissues and neuroepithelium are the first EPO-producing sites in mammalian development.

EPO produced by NEP cells is unable to stimulate yolk sac primitive erythropoiesis up to E9.0 while the embryonic circulation system is under construction (Godin and Cumano, 2005; Palis, 2008), suggesting that erythroid progenitor cells are generated in the yolk sac without EPO signaling until E9.0 (Figure 1). Consistently, the differentiation of hematopoietic stem cells into erythroid progenitor cells is independent of EPO in definitive erythropoiesis (Wu et al., 1995; Suzuki et al., 2003). Soon after the establishment of a stable heartbeat around E9.0, EPO is delivered into erythroid progenitor cells expressing EPOR in the yolk sac and activates the differentiation and proliferation of these cells. Indeed, the levels of phosphorylated STAT5 and its target gene expression are significantly elevated in the E9.0 yolk sac (Suzuki et al., 2013). The bioactivity of NEP-cell-derived EPO was verified by co-incubation of yolk sac cells with the head region of E8.5 embryos. As a result, erythroid cells were expanded in the presence of head cells containing NEP cells, and EPO antibodies neutralized this effect (Suzuki et al., 2013). This evidence indicates that EPO secreted by NEP cells is carried into the yolk sac by the developing circulation system to support primitive erythropoiesis in E9.0 mouse embryos. In fact, mutant mouse embryos suffering from heartbeat defects exhibit the maturation arrest of primitive erythroid cells (Lux et al., 2008).

Since maternal EPO is insufficient for supporting erythropoiesis of Epo-deficient embryos due to blockage in the placenta (Koury et al., 1988; Wu et al., 1995), EPO secreted by NEP cells is considered essential for normal RBC production in developing embryos (Suzuki et al., 2013). As mentioned above, loss of NEP-cell-derived EPO causes the partial defect of primitive erythropoiesis but is not involved in mouse mortality, while hepatic EPO production is essential for survival and the fetal liver definitive erythropoiesis. Importantly, many studies have proposed non-canonical functions of EPO beyond erythropoiesis because EPOR expression is widely detected in embryonic and adult tissues, including neural, endothelial and cardiac cells (Wu et al., 1999; Zhang et al., 2014). Therefore, further studies should investigate whether NEP-cell-derived EPO is required for the development of the neural and circulation systems, using NEP-cell specific EPO-deficient mouse models, the generation of which is technically impossible at this moment.

## Characteristics and Fate of NEP Cells

Most NEP cells are found in the pericapillary microenvironment. There are GFP-expressing trunk neural crest cells in intersomatic regions of the *EpoGFP* transgenic embryos at E9.5, and these cells seem to migrate from dorsal to ventral regions along capillaries (Erickson, 1985; Miller et al., 2010; Suzuki et al., 2013; George et al., 2016; Figure 2C,D). Additionally, neuroepithelial cells expressing the *Epo* gene are found in the neural tube, which is rich in capillaries (Suzuki et al., 2013; Suzuki, 2015; Figure 2B). This spatiotemporal proximity of NEP cells to capillaries is likely a benefit to the effective secretion of EPO into the bloodstream for prompt erythropoietic induction in the yolk sac and bloodstream. Consistent with the biological significance of the pericapillary localization of NEP cells, the site of EPO production shifts to hepatocytes when the sites of erythrocyte maturation in the yolk sac and bloodstream are replaced with the liver during mouse development (Figure 1).

Tracing NEP cells after ceasing EPO production by using another reporter transgenic mouse line, in which cells once having expressed Cre recombinase under *Epo*-gene regulation (the *EpoCre* transgene) are permanently labeled with tdTomato fluorescence, has revealed the wide distribution of NEP-cell-derived cells. For instance, the midbrain, thalamus, pancreas, eyes, and heart contain NEP-cell-derived cells in E15.5 embryos, although these NEP-cell-derived cells no longer produce EPO (Suzuki et al., 2013; Yamazaki et al., 2013; Figure 2E). To understand the molecular characteristics of NEP cells and/or their derivatives, we established an immortalized cell line derived from NEP cell-lineage cells of E15.5 embryos, and the cell line was named “Neplic (*NEP* cell *l*ineage *i*mmortalized and *c*ultivable) cells.” Neplic cells were isolated from the embryonic brain with tdTomato reporter expression and immortalized by infection with a lentivirus expressing SV40 T-antigen (Figure 2F). Neplic cells are positive for WNT1 and Nestin expression, suggesting that these cells are neural lineage cells. However, Neplic cells do not express EPO, HIF2α, GFAP, p75^NTR^, or SOX2. This observation indicates that NEP cells consist of heterogeneous cell types in embryos, and Neplic cells are derived from one of these cell types. It is an attractive hypothesis that NEP cells migrate to developing kidneys and differentiate into REP cells expressing neural genes (Asada et al., 2011; Suzuki et al., 2013).

Hypoxia strongly induces *Epo*-gene expression in hepatocytes and REP cells. However, the effect of oxygen conditions on EPO production in NEP cells remains unidentified due to technical limitations. Since Neplic cells lose HIF2α mRNA expression during maturation, the hypoxic activation of *Epo*-gene expression is invalid in Neplic cells. Notably, the human neuroblastoma cell lines Kelly and SH-SY5Y express the *EPO* gene in a hypoxia-inducible manner (Stolze et al., 2002). Because neuroblastoma is a common pediatric solid tumor originating from the sympathoadrenal lineage of the neural crest, human embryos are expected to have NEP cells secreting EPO for their primitive erythropoiesis. Moreover, these cell lines suggest that NEP cells produce EPO in a hypoxia-inducible manner for the maintenance of oxygen delivery managed by RBCs in embryos.

## Conclusion

Here, the novel function of the neural crest, which is considered the fourth germ layer of vertebrates, has been introduced as the developmentally first site of EPO production. The necessity of the EPO produced by the neural crest for primitive erythropoiesis has been demonstrated, while the roles for EPO in processes other than erythropoiesis are not excluded. In addition, the cell fate and biological significance of NEP cells and the regulatory mechanisms of EPO production in these cells remain unclear. The finding of NEP cells warrants further multi-aspect studies on neural crest cells to unveil the variety of their roles.

## Author Contributions

IH and NS conceived the idea, wrote the manuscript, and generated the figures.

## Conflict of Interest Statement

The authors declare that the research was conducted in the absence of any commercial or financial relationships that could be construed as a potential conflict of interest.
